# Attentional capture by social stimuli in young infants

**DOI:** 10.3389/fpsyg.2013.00527

**Published:** 2013-08-16

**Authors:** Maxie Gluckman, Scott P. Johnson

**Affiliations:** Department of Psychology, University of CaliforniaLos Angeles, CA, USA

**Keywords:** face perception, sex differences, infant development, attention, saliency map

## Abstract

We investigated the possibility that a range of social stimuli capture the attention of 6-month-old infants when in competition with other non-face objects. Infants viewed a series of six-item arrays in which one target item was a face, body part, or animal as their eye movements were recorded. Stimulus arrays were also processed for relative salience of each item in terms of color, luminance, and amount of contour. Targets were rarely the most visually salient items in the arrays, yet infants' first looks toward all three target types were above chance, and dwell times for targets exceeded other stimulus types. Girls looked longer at faces than did boys, but there were no sex differences for other stimuli. These results are interpreted in a context of learning to discriminate between different classes of animate stimuli, perhaps in line with affordances for social interaction, and origins of sex differences in social attention.

Humans are a highly social species. A rich body of evidence highlights the importance of faces to social interactions (Farah et al., 1998; Haxby et al., 2002), and faces are an especially important class of visual stimulus for infants. Building on observations of early developments in visual scanning of faces within the eye region (Haith et al., 1977), researchers have established that faces and face-like configurations attract infants' attention to a greater extent than foil stimuli, as revealed by experiments employing preferential looking (Mondloch et al., 1999), preferential tracking (Johnson et al., 1991) and electrophysiological measures (de Haan et al., 2002) Infants are sensitive to eye contact (Farroni et al., 2002), direction of gaze (Farroni et al., 2004), and emotional expression (Walker, 1982). Infants discriminate between infant- and adult-directed dynamic faces (Kim and Johnson, submitted), and modulate their behavior when engaged in live face-to-face interactions in response to facial movements (Meltzoff and Moore, 1977; Murray and Trevarthen, 1985). These behaviors are subserved by early-developing mechanisms supporting efficient detection and identification of faces and their properties.

The extent to which infants' attention to faces stems from specialized or general-purpose mechanisms is a matter of dispute (e.g., Morton and Johnson, 1991; Valenza et al., 1996; cf. Bukach et al., 2006; McKone et al., 2007). An important approach employed to adjudicate these views addresses the possibility that stimulus attributes unique to faces attract infants' gaze when embedded in complex scenes. Current evidence for this possibility, however, is mixed. On the one hand, when watching a cartoon stimulus, 6-month-old infants' attention was drawn similarly to visually salient regions of the display—defined in terms of motion, color, and luminance—and to faces (Frank et al., 2009), implying that attention to faces is not obligatory, and calling into question the extent to which faces necessarily may be considered a “special” class of stimulus. On the other hand, 6-month-olds' attention was captured more effectively by static faces than by distracters consisting of pictures of common objects arranged in six-item arrays (Gliga et al., 2009; Di Giorgio et al., 2012; see Schietecatte et al., 2011, for a similar demonstration in a more naturalistic setting). Attentional capture persisted when faces were inverted; suggesting that attraction to faces did not likely stem from their configural properties, given the disruptive effects of inversion on face recognition (Turati et al., 2004). Gliga et al. also tested effects of “scrambled” faces to evaluate the possibility that low-level stimulus attributes, present in upright and inverted faces but not in distracters, were responsible for capture; scrambled faces preserved color and contrast of intact faces but disrupted their phase spectra (i.e., featural and configural information). Under these conditions, scrambled faces did not capture attention.

By 6 months, therefore, faces often attract infants' attention in complex scenes, but this effect may not hold across all contexts, and the nature and mechanisms of attentional capture remain unclear. We consider two possibilities. First, faces may attract attention due to an unlearned propensity to orient to face-like visual stimuli, observed in neonates Valenza et al., 1996; Johnson et al., 1991, and perhaps augmented by a stored representation of their social benefits (e.g., affordances for social interactions, importance for identification of conspecifics, and relevance for discriminating humans from other animate entities) that builds over the first several months after birth. On this account, faces may have captured attention in the Di Giorgio et al. (2012) and Gliga et al. (2009) experiments due to a propensity to seek social information. Second, faces may attract attention due to low-level salience of key facial features, most notably the high-contrast eye region, which computational image processing models have established as an especially salient part of the human face (e.g., Li and Ngan, 2008; Khan et al., 2011), and which attracts infants' gaze even in inverted faces (Gallay et al., 2006). On this account, faces captured attention due to an intrinsic propensity to direct gaze toward salient portions of a visual scene (cf. Frank et al., 2009). Note that unlearned mechanisms for detecting faces and eyes (Farroni et al., 2002, 2004) are consistent with each of these accounts, yet play different roles in guiding visual attention: On a face-specific account, an inherent sensitivity to eye contact provides opportunities for social exchange, facilitating acquisition of socially relevant information. On a salience account, attention is drawn to the eye region due to its low-level properties.

As noted, extant evidence from infants is compatible with both face-specific and salience accounts of attentional capture by faces. Evidence from adults, too, is mixed. Experiments have examined the possibility that faces “pop out” preattentively in multi-element arrays, operationalized as response times to detect faces that are independent of the number of distracters. An early attempt to establish facial popout (Nothdurft, 1993) revealed positive evidence when the target was a drawing of an upright face set amongst inverted faces, but the effect remained even when facial features were omitted, implying that the contours of the hairline were responsible. Hansen and Hansen (1988) reported popout of an angry face set amongst happy faces, though this result may have stemmed from luminance differences between target and distracters (Purcell et al., 1996). Kuehn and Jolicoeur (1994) observed facial popout when distracters contained no facial features; face detection was impaired, however, when distracters consisted of rearranged facial features. Likewise, Herschler and Hochstein (2005) found that facial popout was most robust when distracters were visually distinct non-face objects and when inner facial features were clearly visible, either in photographs or drawings, but there was no evidence for popout of other common items, such as cars, houses, or animal faces. Other experiments revealed a processing advantage for faces when adults were asked to detect rapid (“flickered”) changes in item identity (Ro et al., 2001), or to match item identity with a briefly primed category name (Ro et al., 2007), in six-item arrays (faces, appliances, clothing, foods, musical instruments, or plants). The matching study found a similar effect for body parts (e.g., hands), but not for animal faces. In an identification task, however, human and animal faces were both detected rapidly (less than 400 ms) and accurately (greater than 95% correct) when viewed in photographs depicting natural scenes (Rousselet et al., 2003). Inversion impaired performance with human faces to a greater extent than animal faces, consistent with past research demonstrating important contributions of configural information to face recognition (e.g., Farah et al., 1995). Taken together, these studies provide evidence that for adults, as for infants, faces capture attention in complex arrays of distracter objects under some circumstances, yet they fail to reveal the sources of these effects: experience, familiarity, and expertise, or visual properties of the objects themselves.

To examine in greater detail the face-specific account as it pertains to infancy, we investigated 6-month-olds' attentional capture by three types of stimulus. We reasoned that a face-specific propensity to seek social information would lead to rapid attentional capture by full, upright faces, and might extend as well to stimuli that depart to varying extents from this “ideal” stimulus. This included human body parts that were either parts of faces (eyes, noses, and mouths) or were independent of faces (hands and feet). We also examined effects of animals as targets, all of which included faces. Animal faces did not capture adults' attention in a visual search task (Ro et al., 2007), but were detected with brief exposures (Rousselet et al., 2003). Additionally, experience with pets influences infants' visual inspection and categorization of animal pictures (Kovack-Lesh et al., 2008), implying that infants are interested in and learn rapidly about animals. If, however, social stimuli attract attention principally by virtue of their visual salience—perhaps due to regions of high contrast, or combinations of color and luminance—then computer-generated salience maps should reveal that faces and other kinds of social content are typically characterized these visual attributes more than the distracters with which they were paired in our study.

In addition, we addressed the possibility of sex differences in attentional capture by comparing girls' and boys' performance. Connellan et al. (2000) and Alexander et al. (2008) reported greater interest in faces by newborn and 6-month-old girls, respectively, relative to boys, and there is evidence of “female advantage” in face processing in adults, as discussed subsequently. In contrast, Weinberg et al. (1999) reported that 6-month-old boys were more socially oriented than girls when engaged in face-to-face interactions with their mothers. However, the majority of the face perception literature does not report analysis of sex differences, leaving open the question of a female advantage in attentional capture by social stimuli.

We adapted the visual search method first described by Langton et al. (2008) and Ro et al. (2007) with adults and later employed by Di Giorgio et al. (2012) and Gliga et al. (2009) with infants. Infants viewed a series of six-item arrays (Figure 1) in which one item was either a face, body part, or animal. The other five items consisted of a variety of distracters, described subsequently. We observed 6-month-olds due to conflicting evidence at this age for (a) the extent to which visual attention is drawn by faces (Frank et al., 2009; Gliga et al., 2009), and (b) sex differences in social attention (Weinberg et al., 1999; Alexander et al., 2008). We reasoned that attentional capture by social content would be revealed as looking at faces, body parts, and animals at levels greater than might be expected by chance—that is, infants' initial looks at trial onset and overall attention would tend to be directed at the social stimuli. In addition, we obtained salience measures of all stimuli to assess the possibility that the social stimuli may have been the most salient in terms of low-level visual attributes.

## Methods

### Participants

The final sample consisted of thirty-two 6-month-old infants (16 girls and 16 boys), ranging from 5.5 to 6.6 month (*M* = 6.0 month). Five infants were observed but excluded from analysis because they provided data in fewer than half the trials (48 possible). All infants were full-term with no known developmental difficulties. Infants were selected from a public database of new parents and were recruited by letters and telephone calls.

**Figure 1:**
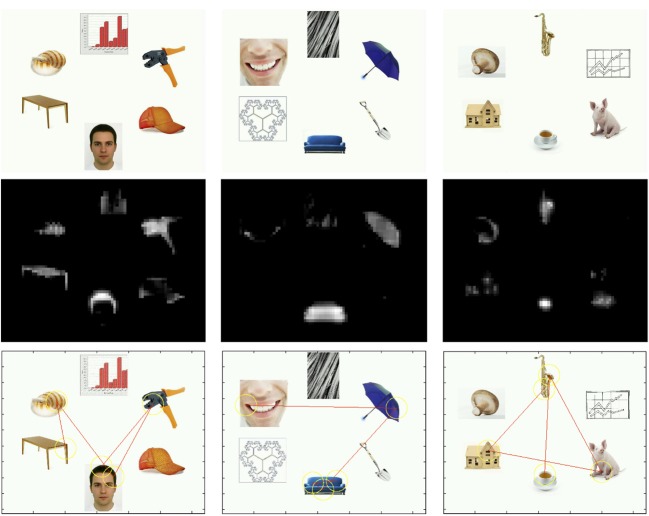
**Top row:** Examples of stimulus arrays with a face (left), body part (center), or animal (right). **Center row:** Saliency maps of stimulus arrays in the top row. **Bottom row:** Modeled visual scanning based on saliency maps. For the face, saliency ranking = 1 (i.e., a region within the face AOI was determined as most salient). For the body part, saliency ranking = 5. For the animal, saliency ranking = 3.

### Apparatus and Procedure

A Tobii 1750 eye tracker with 17-inch monitor (screen resolution 1280 × 1024; refresh rate 60 Hz) was used to collect eye movement data. Stimuli were presented and data collected with Clearview software.

Infants were seated in a parent's lap ~60 cm from the monitor. Each infant's point of gaze was first calibrated with a standard five-point calibration scheme, wherein gaze was directed toward five coordinates on the screen in sequence. All infants provided at least four acceptable calibration points.

Each trial was preceded by an attention-getter to re-center the infant's point of gaze. An experimenter commenced each trial when the infant was determined to fixate on the attention-getter. Following this, infants viewed each stimulus array in turn for 4 s. Infants were tested until completion of 48 trials or until the experimenter determined that the infant's state would not permit further data collection due to excessive fussiness.

### Stimuli

Stimulus arrays consisted of six photographs placed approximately 11.0 cm (10.5° visual angle at the infant's 60-cm viewing distance) from the center of the screen (see Figure 1). Stimulus dimensions varied between ~2.0 and 12.0 cm (1.9–11.4°). Prior to analysis we marked the location and identity of each item with an “area of interest” (AOI) that continued 1 cm (0.96°) past its maximum horizontal and vertical extent.

One “target” photograph in each array contained one of eight faces (two infants, two adult males, four adult females, all presented *en face*), eight body parts (two eyes, three hands, one pair of feet, one nose, one mouth), or eight animals (rabbit, cat, chicken, cow, frog, pig, raccoon, dog). That is, each array contained one target (the face, body part, or animal) for purposes of analysis. The other five distracter items consisted of (a) household items or other artifacts (e.g., battery, globe, lamp, chair, hairbrush), (b) mechanical objects (e.g., bicycle, drill, electric fan, screw), (c) natural objects (e.g., tree, apple, mushroom, seashell), (d) musical instruments (e.g., saxophone, drum, piano, violin) and (e) abstract images or graphs. Photographs were gathered from the internet; each was presented twice over the course of the experiment. One target (face, body part, or animal) was shown during each trial. Stimulus locations were randomly determined. Trial order was pseudorandom such that there were no more than two consecutive trials of each type of target. Trial duration was 4 s.

We used the Saliency Toolbox (www.saliencytoolbox.net) to identify the most salient regions in each stimulus array. The Saliency Toolbox is a set of Matlab functions and scripts that can compute a salience map and model visual scanning based on relative salience of regions within the image (Walther and Koch, 2006). Salience within an image is determined by distinctions in color, luminance, and contour. (Frequency of distinct orientations in an image has been considered a proxy for its complexity; Escalera et al., 2007.) This is accomplished with separate feature maps each tuned to a single elementary attribute in each image (i.e., color, luminance, and contour). These feed into a “winner-take-all” neural network that pools inputs and determines the most salient location by emulating the inhibitory-excitatory organization found in early visual processing (i.e., retinal ganglion cells and the lateral geniculate nucleus), enhancing feature contrast and guiding visual attention. Identification of saliency is not tantamount to detection of objects, instead corresponding a process of highlighting regions of a visual array that may merit attention and further processing by an attentive observer, regardless of acuity, contrast sensitivity, and so forth.

## Results

We conducted three sets of analysis: *first looks, dwell times*, and *salience*. First looks and dwell times correspond to “attention-getting” and “attention-holding” properties of visual stimuli described by Cohen (1972), and analyzed by Gliga et al. (2009). For first looks, the dependent variable was the proportion of trials in which each infant's initial gaze shifts landed in the target AOI (face, body part, or animal). For dwell times, the dependent variable was the accumulation of fixations (expressed in ms) within target AOIs for each infant on each trial. For analyses of first looks and dwell times, we conducted planned comparisons of sex differences for the three target types, or categories of social content (faces, body parts, and animals) separately to ascertain the extent to which girls and boys may have distinct patterns of allocation of social attention. As noted, few studies of social attention report analysis for sex differences, and those that did yielded conflicting results (e.g., Weinberg et al., 1999; Alexander et al., 2008). A second motivation for our approach stems from the possibility of sex differences in social attention to non-face stimuli.

### First looks

**Figure 2:**
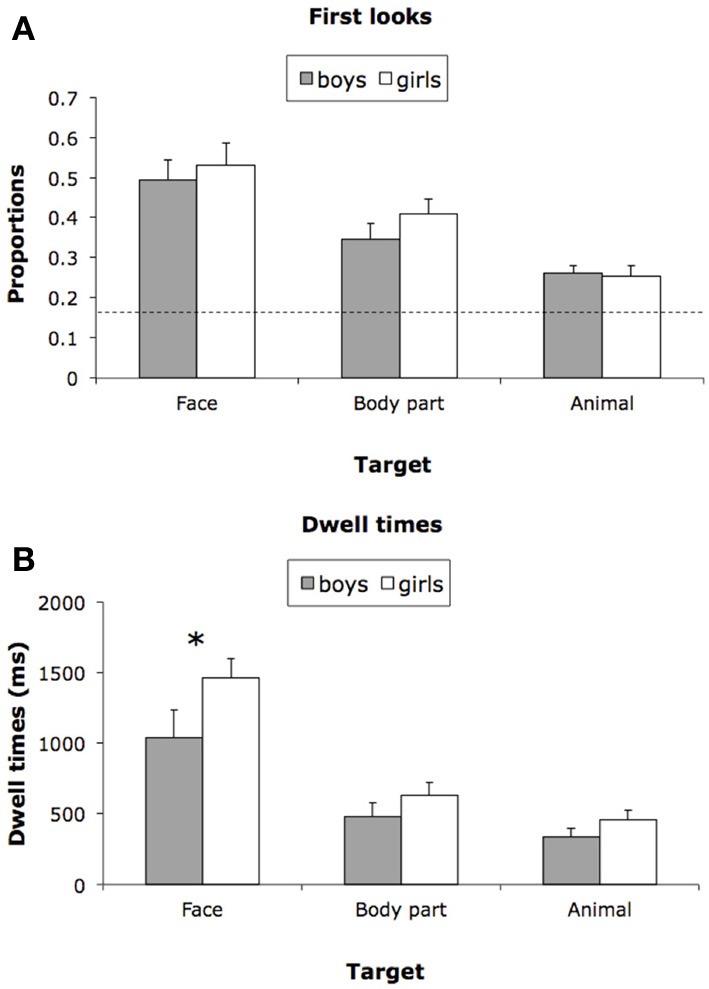
**(A)** *M* first look proportions for girls and boys for each target type; chance level performance = 0.167. **(B)** *M* dwell times for girls and boys for each target type. ^*^p < 0.05.

Figure 2A shows first look proportions for girls and boys for each target type. To address the possibility that targets attract visual attention more than distracters, first look proportions were compared to chance level performance (0.167). First looks toward all three types of target (faces, body parts, animals) were well above chance, *t*s_(31)_ = 9.33, 7.75, and 5.42, respectively, *p*s < 0.0001, *d*s = 3.35, 2.78, and 1.95; first looks toward body parts in faces (eyes, noses, and mouths) were not reliably greater than first looks toward non-face body parts (feet and hands), *t*_(29)_ = 1.56, *ns*. A 2 (Sex: girls vs. boys) × 3 (Target: face, body part, or animal) mixed ANOVA, with repeated measures on the second factor, yielded a significant main effect of Target, *F*_(2, 60)_ = 21.09, *p* < 0.0001, partial η^2^ = 0.41, and no other reliable effects. Tests of simple effects revealed that faces attracted first looks more frequently than body parts, *F*_(1, 30)_ = 8.78, *p* < 0.01, *d* = 0.73, and body parts in turn attracted more first looks than animals, *F*_(1, 30)_ = 16.82, *p* < 0.001, *d* = 0.95. Planned comparisons (simple effects) revealed no sex differences in first looks toward any of the three target types, *F*s < 1.5, *p*s > 0.26, *ns*. No other item category attracted first looks greater than chance levels, and there were no sex differences in first looks to non-social categories, *t*s < 1.5, *p*s > 0.24, *ns*.

### Dwell times

Figure 2B shows dwell times for girls and boys for each target type. A Sex × Target mixed ANOVA yielded a significant main effect of Target, *F*_(2, 60)_ = 60.45, *p* < 0.0001, partial η^2^ = 0.67, and a marginally significant main effect of Sex, *F*_(1, 30)_ = 3.88, *p* = 0.058, partial η ^2^ = 0.11. The interaction was not statistically significant. Tests of simple effects revealed longer dwell times for face stimuli than for body parts, *F*_(1, 30)_ = 57.76, *p* < 0.0001, *d* = 1.22, and longer dwell times for body parts than for animals, *F*_(1, 30)_ = 4.60, *p* < 0.05, *d* = 0.47. Planned comparisons (simple effects) revealed a sex difference in dwell times for face stimuli, *F*_(1, 30)_ = 5.32, *p* < 0.05, *d* = 0.61, but not for body parts or animals, *F*s < 0.2, *p*s > 0.20, *ns*. Dwell times for all other item categories were reliably lower, and there were no sex differences in dwell times for any non-social category, *t*s < 1, *p*s > 0.39, *ns*.

### Salience

Each of the 48 stimulus arrays was processed with the Saliency Toolbox, yielding a salience map and rank ordering of salient regions in the image (see Figure 1 for examples). For each stimulus array we recorded rankings of the most salient regions (a value of 1 = most salient). For faces, *M* rank = 3.31; for body parts, *M* rank =3.63; and for animals, *M* rank = 3.56. For each of the 16 arrays with each type of target (face, body part, and animal), two were the most salient. In other words, targets were the most salient item in terms of low-level visual attributes in only six of the 48 trials (sign test *p* > 0.9999), suggesting that these properties alone, distinct from other stimulus properties (e.g., social affordances), are not likely to have captured infants' visual attention consistently.

## Discussion

We presented six-item arrays of common objects and social stimuli (faces, body parts, and animals) to 6-month-old infants as we recorded their patterns of visual attention. All three types of target attracted infants' attention initially, but face stimuli were best able to maintain attention, especially for girls. These results clarify our understanding of the means by which social content attracts and holds young infants' attention, and they bear important implications for possible sex differences in social development. Each issue is addressed in turn.

Several prominent theories have posited a role for an innate representation of faces, perhaps taking the form of a schematic “template” for facial structure, that guides visual orienting in infants and contributes to formation of cortical mechanisms for face recognition in adults (e.g., Morton and Johnson, 1991; McKone et al., 2007; Sugita, 2009). Such a representation could underlie the effects of face stimuli on the attentional capture we observed. However, it seems unlikely that unlearned representations are the best explanation for similar effects of the other targets, given the number of structural templates (for several distinct body parts and animals) that might be required to account for their attractive properties. We can also rule out a strong contribution from low-level visual attributes (color, luminance, and contour) as being principally responsible for these findings, because faces, body parts, and animals were rarely the most salient stimuli in the multi-item arrays the infants viewed.

In a previous comparison of attentional capture by faces vs. salience in cartoon stimuli, Frank et al. (2009) found no reliable differences attentional capture by faces vs. salience for 6-month-olds, but the stimuli contained motion (of the characters and of background elements as the camera tracked across the scene). Motion can be detected even by very young infants (Banton and Bertenthal, 1997), perhaps due to the relative maturity at birth of low-level motion detection mechanisms in retinal and early cortical areas. Therefore, motion may have been particularly effective in capturing attention at the expense of faces. Gliga et al. (2009) reported infants' attentional capture by upright and inverted but not scrambled faces, concluding that configural aspects of faces are not necessary to attract infants' attention and favoring an account based on faces' color or amplitude spectra. Gliga et al. did not, however, test attentional capture in arrays without faces (nor did Di Giorgio et al., 2012), as we did, nor did they test effects of scrambling other stimulus categories. Therefore, the extent to which color or amplitude spectra can be accepted as the principal determinant of infant performance remains an open question.

Instead, we prefer an account of attentional capture by social stimuli that acknowledges a strong contribution of learning the importance of distinguishing between different animate entities, presumably for their social affordances. Young infants are inclined to respond to all three types of target we tested—faces, body parts, and animals—and such predispositions may form the basis for developmental trajectories promoting optimal social contact with conspecifics and other species. Three independent lines of evidence support this view. First, neonates recognize the correspondence between others' faces and their own (proposed as an important means of differentiating others; Meltzoff and Moore, 1997), and face discrimination skills are refined across the first year after birth through experience-dependent mechanisms (e.g., Pascalis et al., 2002). Second, infants are sensitive to eye contact (Farroni et al., 2004) and recognize aspects of goal-directedness in observed hand motions (Woodward, 1998). Goal detection may have roots in an unlearned capacity to discriminate simple motions of limbs and extremities, such as those that move toward or away from the body (Craighero et al., 2011), and further developments are facilitated by infants' own manual action experience (Sommerville et al., 2005). Third, young infants discriminate animate from inanimate motions (Frankenhuis et al., 2013) and attend to features that differentiate categories of animals (Mareschal and Quinn, 2001). These skills, in turn, may stem from intrinsic biases to attend to animals (Simion et al., 2008) and are facilitated by infants' continued exposure to other species (Kovack-Lesh et al., in press). Taken together, these studies and the present research support a view of development of social preferences built on predispositions to orient toward a limited set of features of animate stimuli, elaborated by the accrual of experience with their defining characteristics. Yet the present results remain compatible with theoretical views of the face as “special,” given their greater attention-getting and attention-holding properties relative to other social content. And our results demonstrate that these properties extend beyond *en face* presentations of wholly visible human faces characteristic of the majority of the literature, including the studies cited here, because the body part and animal stimuli we used contain part-faces in some instances and animal faces, respectively. These effects were diminished, but still greater than for any other stimulus class we tested.

Finally, consider the sex difference in face dwell times that we observed. When sex differences in social attention are reported, they are often consistent with a female advantage (but not always; Pascalis et al., 1998). For example, early reports documented greater interest in infants by women than by men (e.g., Feldman and Nash, 1978; Frodi and Lamb, 1978). More recently, there have been reports of superior performance by females in detection of gaze direction (Bayliss et al., 2005) and face recognition, in particular for female (own-sex) faces (Lewin and Herlitz, 2002), as well as sex differences in hemispheric specialization for face processing (Fischer et al., 2004; Proverbio et al., 2006). The female advantage may have its source in intrinsic differences between girls and boys in spontaneous attraction to faces (Connellan et al., 2000); this, combined with greater exposure to female faces during infancy (Quinn et al., 2002), may help explain the same-sex recognition advantage observed in adults (Ramsey-Rennels and Langlois, 2006).

Notably, however, many published reports of face perception in adults and infants do not mention sex differences. The reasons for this are unknown, but for many such reports, including most cited in this article, no analyses for sex differences are provided. It may be that the methods used in the present study are more sensitive than other paradigms (e.g., ERPs) in revealing performance differences between girls and boys, but this possibility remains speculative. Nevertheless, our results can help clarify the female advantage in social attention seen in adults. Our results reveal no sex differences in initial “attention-getting” attraction to social stimuli, but rather in the ensuing “attention-holding” properties of faces, in particular for girls. By 6 months, therefore, girls and boys are equally liable to show immediate interest in faces, body parts, and animals, but only faces hold girls' interest. Further investigations are necessary to examine the possibility of an “own-sex” face processing advantage in infancy, and the developmental consequences of such an advantage.

### Conflict of interest statement

The authors declare that the research was conducted in the absence of any commercial or financial relationships that could be construed as a potential conflict of interest.
